# Evolving a Healthcare System With a Coordinated Approach for Patient-Reported Measurement of Diagnostic Quality: Comment on "Achieving Diagnostic Excellence: Roadmaps to Develop and Use Patient-Reported Measures With an Equity Lens"

**DOI:** 10.34172/ijhpm.8905

**Published:** 2025-05-18

**Authors:** Karen S. Cosby

**Affiliations:** Center for Quality Improvement and Patient Safety, Agency for Healthcare Research and Quality, Rockville, MD, USA.

**Keywords:** Diagnostic Quality, Quality Measurement, Diagnostic Excellence, Patient-Reported Measures, Patient Safety, Patient-Centered

## Abstract

Quality metrics for improving care are deeply embedded in healthcare systems. Patient-reported measures (PRMs) have now been implemented for many conditions and are a high priority for the Centers for Medicare and Medicaid Services (CMS).^1^ However, the development of PRMs specific to diagnostic quality remains largely exploratory. Early progress in acquiring and analyzing diagnostic PRMs reveals that patients offer a novel and valuable source of information about their diagnostic journeys. To fully understand and learn from patient experiences, work needs to include varied clinical settings, sites, and conditions. This work requires and deserves focused commitment and coordinated effort with a unifying strategic vision optimally facilitated by a national, or international, coordinating center.

## Patients Have Important Information About Their Diagnostic Journey

 Patient-reported measures (PRMs) are an evolving and important source of information about diagnostic quality. However, progress in developing and implementing methods to capture patient feedback about their diagnostic experiences has been slow. There are technical challenges to solve, but there are also obstacles created by medical culture; clinicians may be hesitant to accept laypersons’ conclusions about clinical reasoning and judgment and many may be reluctant to add further burden to quality measurement. To be fair, if PRMs are done well, many might welcome added information, context, and fresh insights patients could provide.

 Barriers to acceptance of diagnostic PRMs can be expected, partly related to a few unstated assumptions that permeate medical culture. While some in healthcare might be reluctant to express certain sentiments, their actions, individually and collectively, reflect the following attitudes or beliefs:

- All relevant information needed for optimal diagnosis is captured in the medical record, and
- All diagnostic activity occurs in discrete encounters within formal structured settings and is accurately recorded.
- Patients often lack expertise in medical science or diagnostic reasoning that limits the value of their input on diagnostic accuracy and quality.
- Surveys and qualitative methods to collect patient feedback may be less trusted and undervalued compared with traditional quality methods using existing healthcare data.

Each of these attitudes is challenged by evidence to the contrary. Generally, medical hubris trusts data in electronic records as the only reliable source of evidence that is accurate and complete, while neither is certain to be true.^2,3^ The adage that “if it isn’t documented, it didn’t happen” might reasonably be supplemented with, “just because it’s documented, doesn’t mean it’s accurate or complete.”

 Independent of any limitation of formal medical records, there is much to be gained by listening to patients. Patients have important information about their symptoms that is not always captured in the electronic health record,and they can provide important information about their diagnostic quality that is distinct and different from what is gained by traditional methods and existing sources of data.^2,4,5^ Patient feedback adds critical information that is both comprehensive and nuanced and can tie all the data points together into a meaningful narrative. Failure to solicit and analyze patient feedback hinders the ability to understand their diagnostic journeys and focus efforts towards improvement.

 Certain information can only be obtained from patients. Patients are the only true source of their lived experience, and they can attest to many of the gaps and communication glitches that delay or complicate their engagement with the health system – information not readily captured in their medical record.^6^ System failures, such as lack of coordination of diagnostic care and miscommunication, are leading causes of diagnostic failure^7,8^ and patients not only have a frontline view but also bear the consequences of system inefficiencies and flaws.As patients navigate the healthcare system, they may experience variable diagnostic trajectories with lengthy delays. Trajectories are particularly challenging for complex problems or rare conditions,although delays in diagnosis are well known for even common problems, especially for those who experience inequities in access to care.^9,10^ A long diagnostic trajectory may drive unnecessary, unfruitful evaluations that may be inconvenient and costly. Significant delays may allow their condition to persist and progress to the point that limits treatment options and even worsens outcome. These delays may be especially impactful for delayed diagnosis of cancers experienced by young adults with colorectal cancer^11^ or sicker patients with comorbid conditions and lung cancer, as examples. Routine institutional assessments of quality, often focused on single clinical encounters using data limited to individual institutions and settings, may not identify these potentially preventable problems, and thus fail to design system interventions that could improve timely evaluations – problems and interventions that would be informed by patients.

 PRMs are in wide use for many conditions, most notably to monitor surgical outcomes and track metrics for oncology care, however efforts to collect patient measures to monitor diagnostic quality are in a nascent stage. Interesting and important foundational work is beginning to define methods and approaches to learn about patient’s diagnostic experiences.^12-15^ While patients may not use the same medical jargon that aligns with healthcare professionals, they can convey if their diagnosis made sense, was effectively communicated, and addressed their needs. Information from patients could provide opportunities to react and correct course when patients have reason to suspect that their medical record is wrong, their diagnosis fails to make sense, or when they get lost in their diagnostic journeys. Lessons from PRMs could inform system design to make processes more timely, efficient, and patient-friendly. Patient reports can support the growing paradigm shift in patient-centric care where patients are partners in reaching their diagnoses. Only they experience their illness and fully understand the impact on their lives. And they are the ones with the greatest to lose when diagnostic errors directly lead to prolonged suffering or disease progression with potential for worse outcomes.

## Coordinated Efforts to Design and Implement Meaningful and Impactful Diagnostic Patient Reported Measures

 McDonald et al^16^ list goals that can be achieved with PRMs for diagnosis, among them the ability to learn about and react to diagnostic delays, connect with patients to earn their trust, capture information for quality improvement, and inform research for improving diagnosis – all purposes that support a learning healthcare system to drive diagnostic excellence.

 Having made the argument that patients can contribute to a better understanding of their disease and their diagnostic journeys, the obvious questions are “how,” “when,” “for whom,” and “for what purpose.” The questions are simple enough, but diagnostic errors and their solutions are complex. To be successful, measurement needs to be specific. But diagnostic plans are often variable and numerous, and no single measure is likely to leverage action that guarantees overall diagnostic success that is scalable and generalizable. Successful work to use patient reports to improve diagnosis needs a strategy across the continuum of care that may eventually require multiple sampling points at critical points of action. Human centered design and implementation science is needed to assess the effectiveness of measurement approaches and establish priorities for measurement targets. The essential work is broad and expansive, and without overarching goals and coordinated efforts, there is a risk that siloed efforts may sputter and have limited penetration across health systems. Success can be enhanced if driven by a collaborative community that is aligned by a uniform strategic vision. Such work needs coordination across the healthcare system.

 The paper by McDonald et al^16^ offers a framework for roadmaps to coordinate efforts over time to move the field forward towards strategic goals and milestones. This framework would recognize synergies across different measurement targets and tackle common challenges such that lessons learned from one project could inform others. Rather than struggle to define a single diagnostic target assessed at a moment in time or phase of work as an isolated metric for diagnostic quality, this framework could foster collaboration and coordination of overall efforts and resources for success. The roadmaps provide a high-level perspective to achieve a robust infrastructure to support a vision for a “patient-reported diagnostic excellence measurement system.” Their comprehensive and long-range approach provides a valuable starting point for discussion.

## A Vision for Diagnostic Excellence Informed by Patient Reported Measures

 PRMs have proven successful in improving outcomes for many conditions and procedures. While diagnosis is particularly challenging, there is much to be gained in advancing patient reports for diagnostic excellence. Effective, coordinated work to design and implement methods to capture information from patients could provide a modernized understanding of early disease manifestations and advance the science of diagnosis. One attractive priority target might be to improve the recognition of early symptoms of cancer and strive to improve the stage of disease at time of diagnosis. Defining and measuring key points in the diagnostic process that are most vulnerable to failure could help prioritize improvement efforts where they matter the most. Measurement could inform the development of processes and pathways for common diagnoses where patients and their data tend to be lost and help design streamlined common pathways for reliable and smooth workups. Measurement of diagnostic processes can help benchmark new standards for diagnostic quality, such as condition-specific guidelines for timeliness and efficiency.

 The vision of a global, comprehensive approach to diagnostic PRMs, led and organized by an independent and authoritative center that can convene experts, debate methods and priorities; analyze and summarize lessons, disseminate best practices and tools, and coordinate global efforts is an exciting model that could synergize efforts, optimize impact, and make best use of available funding and resources. The challenge, as always, is to attract and support the talent and sustain a long-term strategic vision to benefit all. This will likely require private-public partnerships with aligned priorities. The roadmap model proposed by McDonald is an ambitious and valuable beginning to that vision.

## Ethical issues

 Not applicable.

## Conflicts of interest

 Author declares that she has no conflicts of interest.

## Disclaimer

 The views expressed in this paper are those of the author and do not necessarily reflect the official policy or position of the United States government or any agency thereof.
